# Insights into predicting small molecule retention times in liquid chromatography using deep learning

**DOI:** 10.1186/s13321-024-00905-1

**Published:** 2024-10-07

**Authors:** Yuting Liu, Akiyasu C. Yoshizawa, Yiwei Ling, Shujiro Okuda

**Affiliations:** Medical AI Center, Niigata University School of Medicine, Niigata City, Niigata 951-8514 Japan

**Keywords:** Retention time prediction, Liquid chromatography, Untargeted metabolomics, Small molecules, Deep learning, QSRR, SMRT, MassBank, PredRet, RepoRT

## Abstract

**Abstract:**

In untargeted metabolomics, structures of small molecules are annotated using liquid chromatography-mass spectrometry by leveraging information from the molecular retention time (RT) in the chromatogram and *m/z* (formerly called ''mass-to-charge ratio'') in the mass spectrum. However, correct identification of metabolites is challenging due to the vast array of small molecules. Therefore, various in silico tools for mass spectrometry peak alignment and compound prediction have been developed; however, the list of candidate compounds remains extensive. Accurate RT prediction is important to exclude false candidates and facilitate metabolite annotation. Recent advancements in artificial intelligence (AI) have led to significant breakthroughs in the use of deep learning models in various fields. Release of a large RT dataset has mitigated the bottlenecks limiting the application of deep learning models, thereby improving their application in RT prediction tasks. This review lists the databases that can be used to expand training datasets and concerns the issue about molecular representation inconsistencies in datasets. It also discusses the application of AI technology for RT prediction, particularly in the 5 years following the release of the METLIN small molecule RT dataset. This review provides a comprehensive overview of the AI applications used for RT prediction, highlighting the progress and remaining challenges.

**Scientific contribution:**

This article focuses on the advancements in small molecule retention time prediction in computational metabolomics over the past five years, with a particular emphasis on the application of AI technologies in this field. It reviews the publicly available datasets for small molecule retention time, the molecular representation methods, the AI algorithms applied in recent studies. Furthermore, it discusses the effectiveness of these models in assisting with the annotation of small molecule structures and the challenges that must be addressed to achieve practical applications.

**Supplementary Information:**

The online version contains supplementary material available at 10.1186/s13321-024-00905-1.

## Introduction

In untargeted metabolomic analysis, the most reliable method for metabolite annotation is to compare the chromatographic retention times (RTs) and mass spectral fragmentation patterns of compounds with those of standard substances. However, these standards may be expensive for individual laboratories, and their annotation coverage is often low. Therefore, initial annotation of unknown compounds in silico can provide a theoretical basis for their identification. The most direct approach to identify candidate compounds is to search the experimental spectrum against mass spectral libraries. Owing to the limited annotation of compounds not in the database, further efforts are needed, such as the prediction of in silico structures or spectra using algorithms.

Machine learning (ML) algorithms, which are based on statistical models and learn from large datasets, and their sub-branch deep learning (DL), which excels at extracting complex data features using neural networks, have been applied for various purposes, including data mining, image recognition, and prediction analysis [1]. In mass spectrometry (MS), CSI-FingerID [2] employs algorithms such as multiple kernel learning and support vector machines (SVM) to predict fingerprints for molecular structures. By integrating with SIRIUS 4 [3], the approach evaluates the best match by comparing the scores generated from the predicted fingerprints, derived from spectrum-computed fragmentation trees, against the fingerprints generated for each structure in the database. In addition, ML algorithms are used to generate in silico reference spectra for small molecules, thereby extending the coverage of mass spectrum libraries [4, 5]. As of 21 Feb, 2024, there were 1844353 in silico data covering 89% of the spectra in the MoNA database [6]. Moreover, the application of DL algorithms has led to the generation of in silico compound structures from mass spectra, as demonstrated by tools like MSNovelist [7] and MassGenie [8]. However, MS data often correspond to numerous candidate compounds that share the same molecular mass and exhibit similar mass spectral fragmentation patterns.. The RT information from liquid chromatography (LC), which correlates significantly with the molecular structure, can serve as a filter to eliminate false positives, significantly narrowing the range of candidate compounds and resulting in a more accurate annotation.

Common molecular separation methods include reversed-phase (RP), hydrophilic interaction LC (HILIC), and normal-phase (NP) separation. RP uses non-polar particles, such as octadecyl-modified silica particles (C18 column), and a polar liquid phase. These nonpolar particles strongly attract nonpolar molecules, such as hydrocarbons, including aliphatic and aromatic compounds, making them widely used in the separation of secondary metabolites. In contrast, HILIC has polar particles in a nonpolar liquid phase. Its polarity increases with the addition of water, making it suitable for the separation of hydrophilic compounds that are weakly retained in RP. Therefore, depending on the target compound, the separation methodology must be adjusted to suit the purpose of the separation. No single method can comprehensively cover all the types of compounds.

In Nicoud's book [9], the chromatographic equation is described. By integrating Eqs. 1.22, 1.27, 1.31, and 1.7 mentioned in the book, a rough calculation formula for RT can be obtained using Eqs. 1 and 2:

1 $$\varvec{t_{R} \, = \,\frac{1}{Q}\left( {V + \overline{V} \cdot \overline{{K_{A} }} } \right)}$$

2 $$\varvec{ \overline{{K_{A} }} = \varepsilon_{i} + \left( {1 - \varepsilon_{i} } \right) \cdot \widehat{{K_{A} }}}$$

where ${\varvec{Q}}$ represents the flow rate, which is typically measured in mL/min. ${\varvec{V}}$ denotes the volume of the extragranular fluid within the column while $\overline{{\varvec{V}} }$ refers to the volume of the lumped solid phase within the column. The lumped solid phase included the skeleton of the beads and fluid present in the intragranular pores. The term ${{\varvec{\varepsilon}}}_{{\varvec{i}}}$ represents the internal porosity of the column, and accordingly, $1-{{\varvec{\varepsilon}}}_{{\varvec{i}}}$ signifies the intragranular porosity. $\overline{{{\varvec{K}} }_{{\varvec{A}}}}$ is the lumped Henry coefficient related to $\widehat{{{\varvec{K}}}_{{\varvec{A}}}}$. $\widehat{{{\varvec{K}}}_{{\varvec{A}}}}$ is the standard Henry’s coefficient, which varies with factors, such as the temperature and solvent composition. Essentially, $\overline{{{\varvec{K}} }_{{\varvec{A}}}}$ and $\widehat{{{\varvec{K}}}_{{\varvec{A}}}}$ are measures of the affinity of the solute for the stationary phase relative to the mobile phase. These parameters collectively suggest that the RT in chromatographic processes can be influenced by a variety of column parameters. These parameters include the material used for column filling, capacity, porosity, column temperature, solvent composition, solvent gradient, and flow rate. Each of these factors can significantly affect the interaction between the analyte and stationary phase, thereby altering the RT of different compounds as they pass through the column. Owing to the factors mentioned above, the RT measured by different LC systems can exhibit significant variability. This variability creates challenges when using RT information for metabolite annotation across different experimental platforms. Therefore, developing methodologies to accurately predict the RT of small molecules in different LC systems can facilitate the application of in silico metabolite annotation to laboratory-specific chromatographic systems.

This review primarily focuses on the research progress related to the prediction of LC RTs. It includes discussions on publicly available data sources and molecular structure representation, and particularly emphasizes the application of AI technology in RT prediction methodologies, especially in the context of the large METLIN small-molecule RT dataset released in the last 5 years.

## Datasets with liquid chromatography RTs

Spectrum databases include freely available library HMDB [10], GNPS (Global Natural Products Social Molecular Networking) [11], ReSpect (RIKEN MSn spectral database for phytochemicals) [12], MassBank [13], MoNA [6], METLIN [14], SDBS (Spectral Database for Organic Compounds, AIST) [15] and commercial library NIST23 (NIST Mass Spectral Libraries, 2023 Edition) [16], METLIN Gen2 [17], mzCloud [18] as well as Wiley-MSforID [19]. Comparison between partial free and commercial spectrum libraries is well reviewed by Vinaixa et al. [20]. In addition to organized spectrum databases, spectrum is also possible to be generalized from raw data databases Metabolights [21] and Metabolomics Workbench [22]. In this review, we focus only on open-access data sources.

RT data are recorded relatively less frequently than mass spectrum data in large libraries. The HMDB, GNPS, ReSpect, and SDBS libraries only provide MS spectrum data without RT information. The Open-source METLIN only provides spectrum search services and neither spectrum data nor RT information downloads. MassBank and MoNA have MS spectral data, some of which contain additional RT information and maybe useful sources for integrating spectrum and RT. MassBank provide detailed and structured information about chromatography system, MS peak list and RT. It's worth noting that, although partial datasets in MoNA provide submitter name, submitter institute, submitter email, column name and instrument name, basically they do not provide information about eluents, gradient etc. For more rigorous considerations for using RT data in MoNA, it is better to contact submitter to confirm if the data is measured under same chromatography system. Except for MassBank and MoNA, the Metabolights and Metabolomics Workbench libraries are good candidate sources for acquiring RT datasets, which collect raw experimental spectrum data, including target and untargeted metabolite profiling of animals, plants, and microorganisms under various treatments from public studies. In theory, spectra and RTs can be summarized as large datasets by reusing the raw data. Metabolite annotation in the Metabolights library is a hybrid of manual, chemical standard, and software automated annotation. Therefore, studies with convincing annotation (authentic chemical compound-supported annotation) are a better choice for training.

In addition to spectral libraries, datasets published in articles were the primary complementary data sources. The METLIN small molecule RT (SMRT) dataset [23] is the first large RP chromatography RT dataset of 80038 standardized molecules measured under a unified system that has been widely used as a training dataset since its publication. In addition to SMRT, another recently publicized largest machine-learning ready dataset RepoRT [24] collate information from 373 datasets, providing 88325 RT entries covering 49 different LC systems through highly rigorous error correction. In addition to these two large datasets, chiral molecule RT (CMRT) dataset, contains RT information for 25847 (11720 pairs) chiral molecules detected by 25 column systems in 644 reports, which is expected to expand the data sources for model training [25]. Besides large-scale organized datasets, RT information (usually no more than 1000) is provided in additional files for several articles [26–31]. Portions of these experimental data were organized to PredRet [32], which provides an easy method for reuse, and they have been downloaded by subsequent researchers as common small datasets for transfer learning or model evaluation [23, 33–37]. PredRet has maintained a certain level of data acceptance over the past 8 years and has received 287 experimental chemical datasets with 32463 data records shared by users until 11 Nov, 2023. One thing to keep in mind is that the uploaded systems may have uncertain or suspicious data for their open properties without supervision. If the uploaded data records are collected for training or evaluating the model, they should be distinguished with caution, and reliable data sources should be chosen. It's worth mentioning that Predret sources are also integrated into RepoRT dataset through their procedurally processing.

**Fig. 1:**
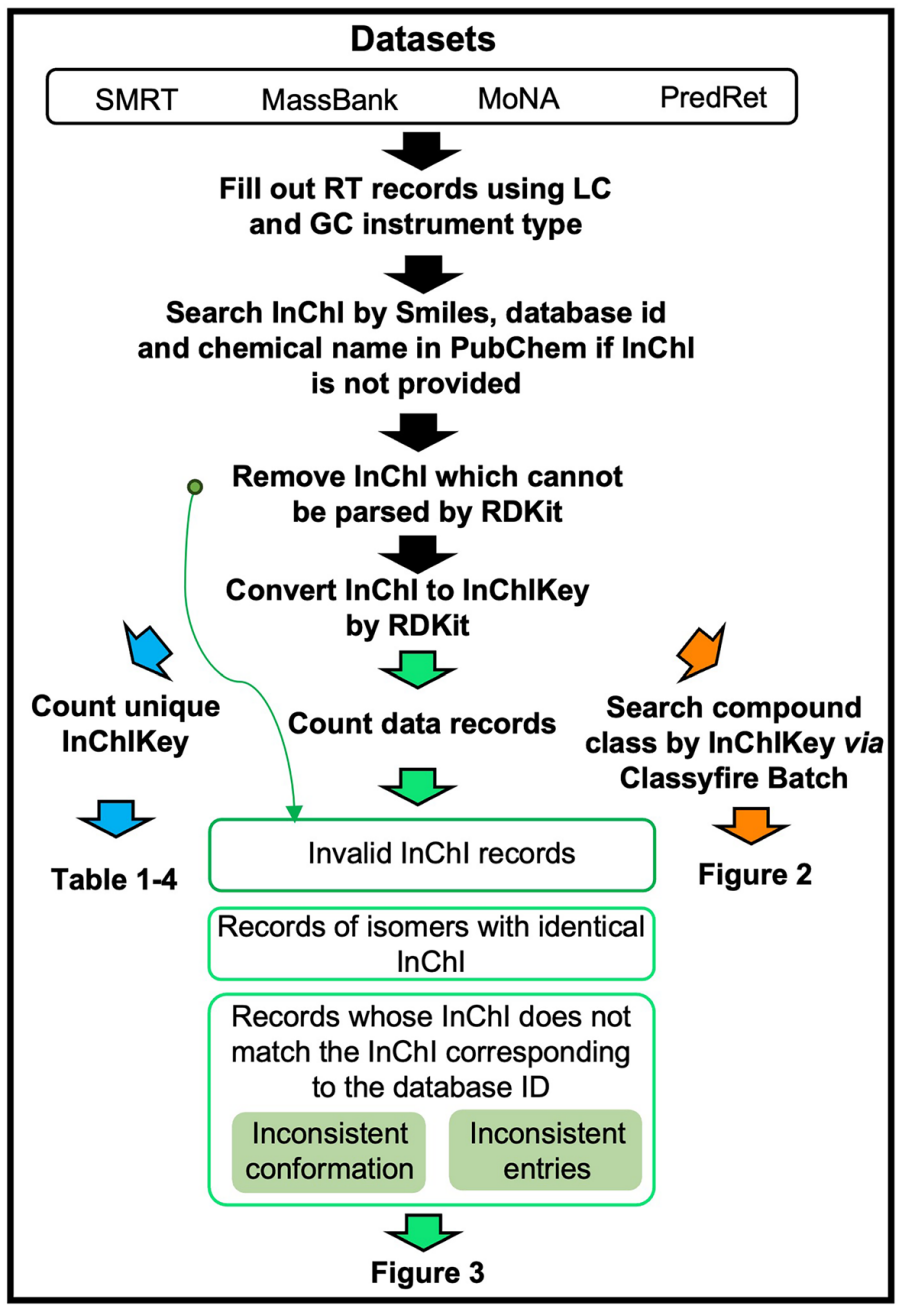
Workflow of the evaluation of retention time records using the SMRT, MassBank (release version Nov, 2023), MassBank of North America (MoNA; accessed on 11 Nov, 2023), and PredRet (accessed on 11 Nov, 2023) databases in this review

**Fig. 2:**
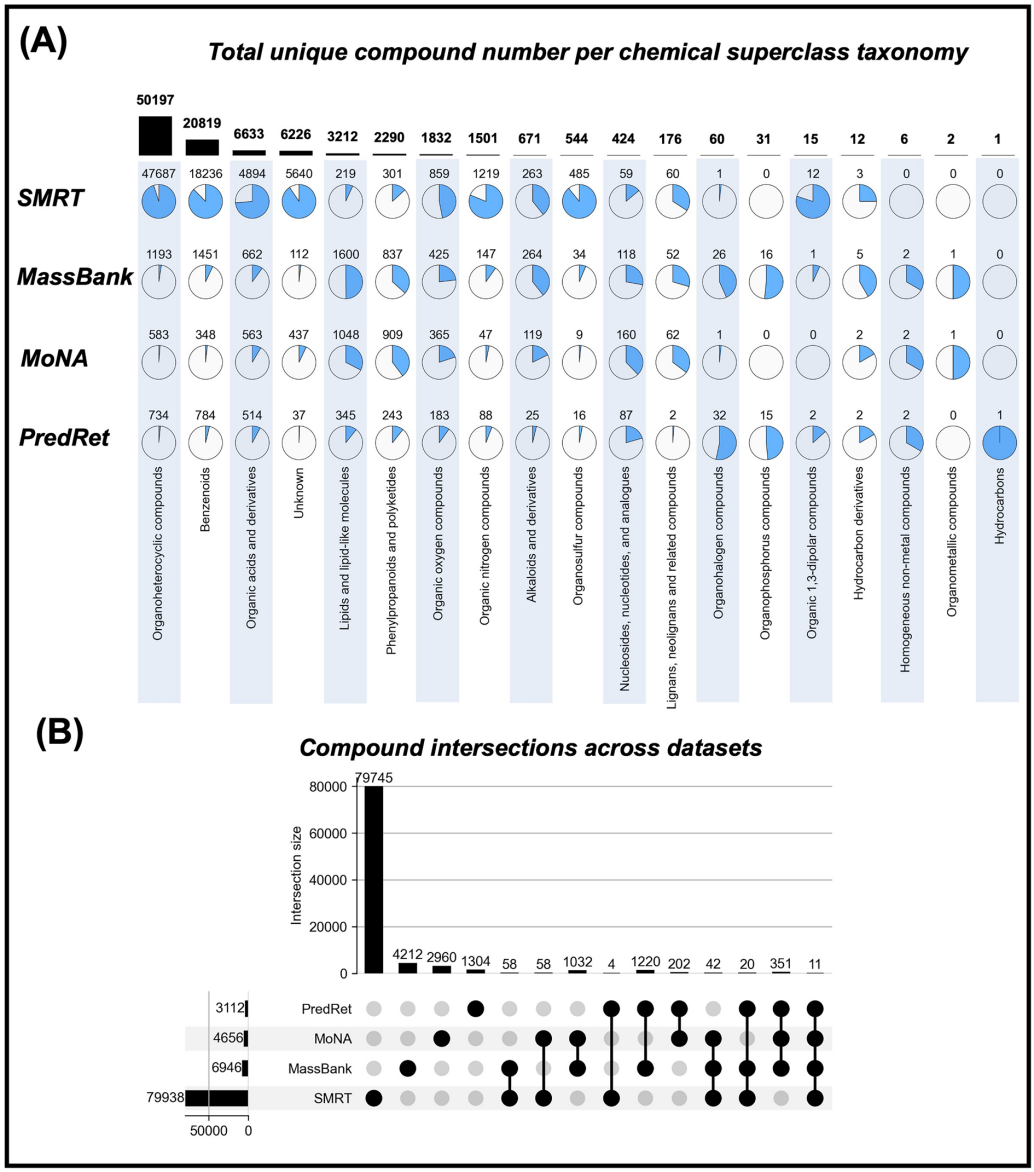
Overview of the liquid chromatography retention time records obtained from the SMRT, MassBank, MoNA and PredRet databases. **A** Ratio of unique compound numbers measured using liquid chromatography across datasets at superclass taxonomy level; compound classes were identified in the Classyfire Batch [39] by searching the International Chemical Identifier key (InChIKey). **B** Compound intersection numbers across the four datasets. Repeated compounds were removed in each dataset

To the best of our knowledge, no studies have collated and described the important datasets, SMRT, MassBank, MoNA, and PredRet as a whole. Therefore, we organized the numbers of data records that can be used for RT prediction across these main datasets in Tables 1, 2, 3, and 4, following the workflow in Fig. 1. In total, 81167 LC data records and 1761 GC data records out of 117966 entries in MassBank (Nov, 2023 updated version), 30981 LC data records and 44 GC data records (excluding MassBank sources) in MoNA (Accessed on 11 Nov, 2023), and 6067 LC data records in PredRet (Accessed on 11 Nov, 2023) provided valid molecular chemical identifiers and RT information (Table 1). Because model evaluation and transfer learning often use data from the same LC system, the RT records were also counted from independent data sources in each large dataset. Moreover, 33 LC data sources and 5 GC data sources in MassBank (Table 2), 20 LC data sources and 3 GC data sources in MoNA (Table 3), and 20 LC data sources in PredRet (Table 4) provided useful RT records.In terms of compound class coverage, 18 of 26 organic chemical taxonomies at the superclass level were covered across four datasets, SMRT, MassBank, MoNA, and PredRet, by searching the InChIKey identifier in the ClassyFire Batch website application [39]. Figure 2A shows the proportions of the four datasets for each chemical classification superclass. The numbers of intersecting compounds across the four datasets are shown in Fig. 2B. MassBank, MoNA, and PredRet all contained specific compounds that were not included in the SMRT dataset, indicating that they are suitable for evaluating the transfer ability of RT prediction models trained on SMRT.

**Table 1:** Investigation of liquid chromatography records with retention time information and valid molecular identifiers in datasets^a^

Datasets	Total records	RT records (LC)	Number of unique compounds (LC)^d^	RT records (GC)	Number of unique compounds (GC)^d^
SMRT	80038	80038	79938^e^	0	0
MassBank	117966	81167	6946	1761	726
MoNA	210156	30981^b^	4656	44	26
PredRet	32463	6067^c^	3112	0	0

^a^See Fig. 1 for the workflow of the survey. The ''total records'' indicated total entry number in libraries. ''RT records (LC)'' indicated the number of entries which instrument type can be recognized as liquid-chromatography instrument by matching ''LC'' in name (e.g. LC-ESI-QTOF), same with ''RT records (GC)'' which could be recognized as gas-chromatography instrument by matching ''GC'' in name (e.g. GC-EI-QqQMS). Record entries which did not indicate instrument type or measured by MS instrument (e.g. QTOF) only were excluded from total entries for subsequent analysis

^b^The 30,981 records did not include records from the MassBank data source to prevent duplicate analyses of the same records in MassBank

^c^6067 records included sources with DOIs but did not include records from MassBank sources to prevent duplicate analyses

^d^The method for counting unique compounds was based on the International Chemical Identifier keys (InChIKeys) as previously described [20]. InChIKeys were generated from identifiers based on the priority order of InChI, SMILES, PubChem CID, KEGG ID, CAS ID, ChEBI ID, or IUPAC name identifiers, stereo information was not excluded if it was provided. Records in which InChI could not be parsed using RDKit (v2023.09.04) [38] were excluded from analysis

^e^81 invalid InChI identifiers and 36 duplicated InChI from 17 pairs stereoisomers with same InChI identifiers and InChIKey, were observed

**Table 2:** Statistical analysis of analytes in the MassBank database using the defined method shown in Fig. 1

MassBank source	Compound number	Unique compound number per data source
Liquid chromatography		
RIKEN	10383	1249
BAFG	19783	1129
Eawag	13210	1055
Athens_Univ	5158	868
LCSB	5582	783
Waters	2719	519
Washington_State_Univ	2626	489
CASMI_2016	622	481
Chubu_Univ	2185	453
UFZ	3154	437
Antwerp_Univ	1762	309
RIKEN_IMS	754	301
BS	1253	291
BGC_Munich	903	223
Eawag_Additional_Specs	748	184
HBM4EU	2317	171
AAFC	950	149
GL_Sciences_Inc	174	147
Univ_Toyama	253	140
NaToxAq	3756	130
ACES_SU	271	108
MPI_for_Chemical_Ecology	691	102
Fukuyama_Univ	340	89
NAIST	621	74
KWR	207	55
PFOS_research_group	413	54
MetaboLights	58	48
IPB_Halle	79	39
UoB	37	37
MSSJ	130	27
CASMI_2012	23	11
UPAO	2	2
Osaka_MCHRI	3	1
Gas chromatography		
Athens_Univ	475	96
Kazusa	273	163
MSSJ	323	175
Osaka_Univ	449	357
RIKEN	241	194

Unique compounds were individually counted based on the International Chemical Identifier key (InChIKey) for each data source

**Table 3:** Statistical analysis of analytes in the MassBank of North America (MoNA) database using the defined method shown in Fig. 1

MoNA source	Compound number	Unique compound number per data source
Liquid chromatography		
Vaniya/Fiehn Natural Products Library	9416	1577
Fiehn HILIC Library	3059	1218
RIKEN PlaSMA Authentic Standard Library	8655	586
QiaoLab_PGN	1329	557
EMBL-MCF	1293	431
Gunma university	3438	402
MetaboBASE	1253	289
US Meat Animal Research Center	364	274
IISPV, URV, CIBERDEM-ISCIII, and UCDavis	387	78
BOKU	215	76
University of Minnesota	1441	52
Uppsala University	70	34
UC Davis	26	25
University of California, Davis	5	5
University of Illinois at Chicago	5	5
Frau	3	3
Weber Flavors	3	3
Institute of Physiology of the Czech Academy of Sciences	15	2
MIT	1	1
University of Antwerp	3	1
Gas chromatography		
Osaka University	23	20
HMDB	20	5
Weber Flavors	1	1

Unique compounds were individually counted based on the InChIKey for each data source

**Table 4:** Statistical analysis of analytes in the PredRet database using the defined method shown in Fig. 1

PredRet source	Compound number	Unique compound number per data source
Liquid chromatography		
BfG_NTS_RP1	912	907
Bade_Publi	1582	675
Cao_HILIC	602	509
RIKEN	469	421
FEM_long	420	412
KI_GIAR_zic_HILIC_pH2_7	538	399
Eawag_XBridgeC18	364	364
Waters STA Forensic	264	220
LIFE_old	194	183
LIFE_new	184	173
IPB_Halle	82	76
FEM_short	72	72
CBM_TEST_F	122	59
Chen_Waters_SERI2019_58PFAS	58	58
CBM_Test_G	102	51
MTBLS4	34	34
MTBLS20-LIUMIN	29	22
WORKPJ	18	18
MPE_IPK_Gatersleben	12	11
semitargetedHSST3	9	8

Unique compounds were individually counted based on the InChIKey for each data source

### Discussion about representations in small molecular RT datasets

**Fig. 3:**
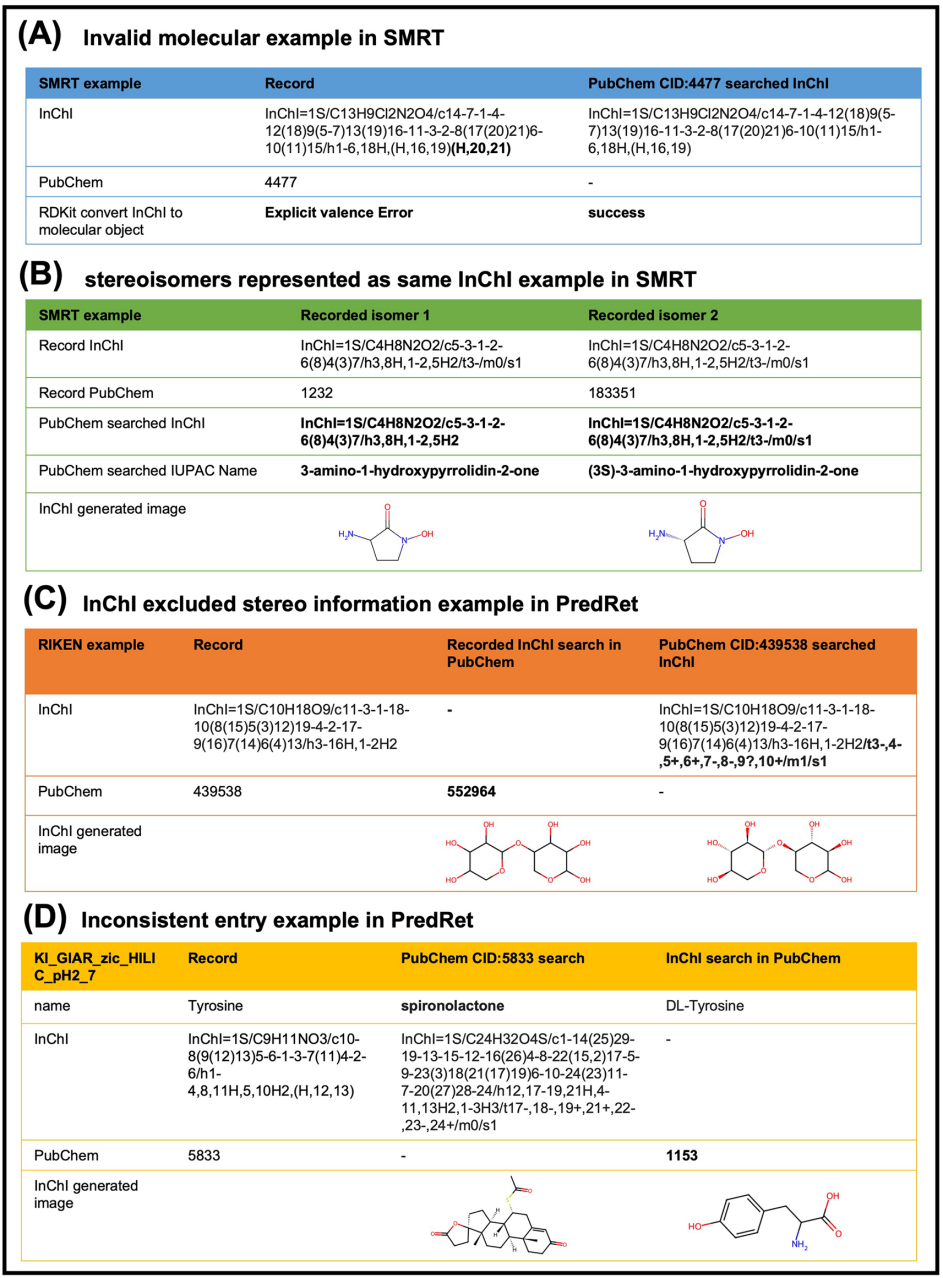
Examples of four discrepancy types. Cases in (**A**) can be adjusted by searching for the PubChem identifier. Stereoisomers in SMRT datasets with different RTs shown in (**B**) are represented by the same InChI and same structural information in the SDF file; however, they can be distinguished by searching PubChem identifier, depending on the researcher's discretion. **C** PredRet strips stereo information for projection methods, and the structure therefore does not always match the reported PubChem entry, which also depending on the researcher's discretion. **D** Partial entries within individual dataset in PredRet may refer to different molecular objects and need to be carefully verified if they are to be used

While working with the dataset, we observed several inconsistent cases in the representations of molecules, in which the same molecule's InChI, database ID, and name were not match. This phenomenon was characterized by comparing the chemical identifiers (InChI, database ID, and nomenclature) denoting compound entities. We categorized and discussed these discrepancies into four types as illustrated in Fig. 3**.** We exemplify type 1 and type 2 by using the SMRT dataset (Table 5). This dataset provides PubChem CIDs, InChI identifiers, and SDF files containing structural information for molecular representation. Type 1 included the cases that InChIs could not be converted into valid molecules using RDKit [38], with 81 entries in SMRT. By searching the InChI correspongding to recorded PubChem CID, successful conversions were achieved (Fig. 3A). Type 2 involved identical representations (InChI and structural information in SDF file) for stereoisomers which exhibit different RTs in SMRT. By searching the InChI corresponding to recorded PubChem CID, the representations could be distinguished **(**Fig. 3B**)**. Whether or not this type is treated specifically depends on the researcher's considerations regarding the representation of stereoisomers. Type 3 and type 4 were exemplified by using PredRet dataset (Table 5). A subset of 32 datasets (listed in Supplementary Table 1), selected from a total of 287 experimental small datasets possessing digital object identifiers (DOIs) and did not cover by MassBank database, was employed for counting cases for type 3 and type 4. Of the 6185 records in these 32 datasets, 5638 records with InChI and PubChem CID or nomenclature were assessed. Type 3 involved cases that recorded InChI differed from PubChem CID searched InChI in terms of stereo information (1660 entries). PredRet strips stereo information in InChI lead to more overlapping compounds between systems, therefore the structure does not always match with reported PubChem entry as shown in Fig. 3C. Typically, the discrimination of enantiomers needs specialized columns; thus, except for diastereomers, records excluding stereo information normally exert minimal influence on RT prediction models intended for application in contexts that utilize standard columns such as C18 or T3. Type 4 involved cases that recorded InChI and PubChem CID referred to completely different molecular object (788 entries, Fig. 3D) which required carefully verification if they are to be used.

**Table 5:** Discrepancy records in datasets

Dataset	Record type			
	Invalid molecular object	Indistinguishable InChI of stereoisomers	InChI excluded stereo information	Inconsistent entries
SMRT	81	36^a^	Not analyzed	Not analyzed
PredRet	78	Not analyzed	1660^b^	788^c^

^a^Pubchem IDs for stereoisomers are distinguishable

^b^Characterized by matching converted InChI identifiers unified by excluding stereo information using RDKit for PubChem ID/ nomenclature searched InChI and recorded InChI

^c^Includes 36 invalid molecular objects

## AI-driven developments in the field of quantitative structure-retention relationship (QSRR)

There are two main methodologies that current studies attempt to address: (1) making efforts toward to developing an accurate RT prediction methodology using QSRR calculations, and (2) unifying the shift of RT using the projection method. Projection methods are introduced in Section ''Development of RT projection methodology for metabolite annotation''. QSRR is a field that has been developed over a long period of time since 80s, and the traditional process consists of molecule description, feature selection, and model construction, with specific categories and related software in the detailed description in the 2018 review [40], and the conceptualization of QSRR in RP, HILIC and IC is described in detail in the 2020 review [41]. Herein, we focus on the development of the QSRR field driven by AI technologies that have emerged with the publication of a large training dataset for SMRT.

### Molecular representation

**Fig. 4:**
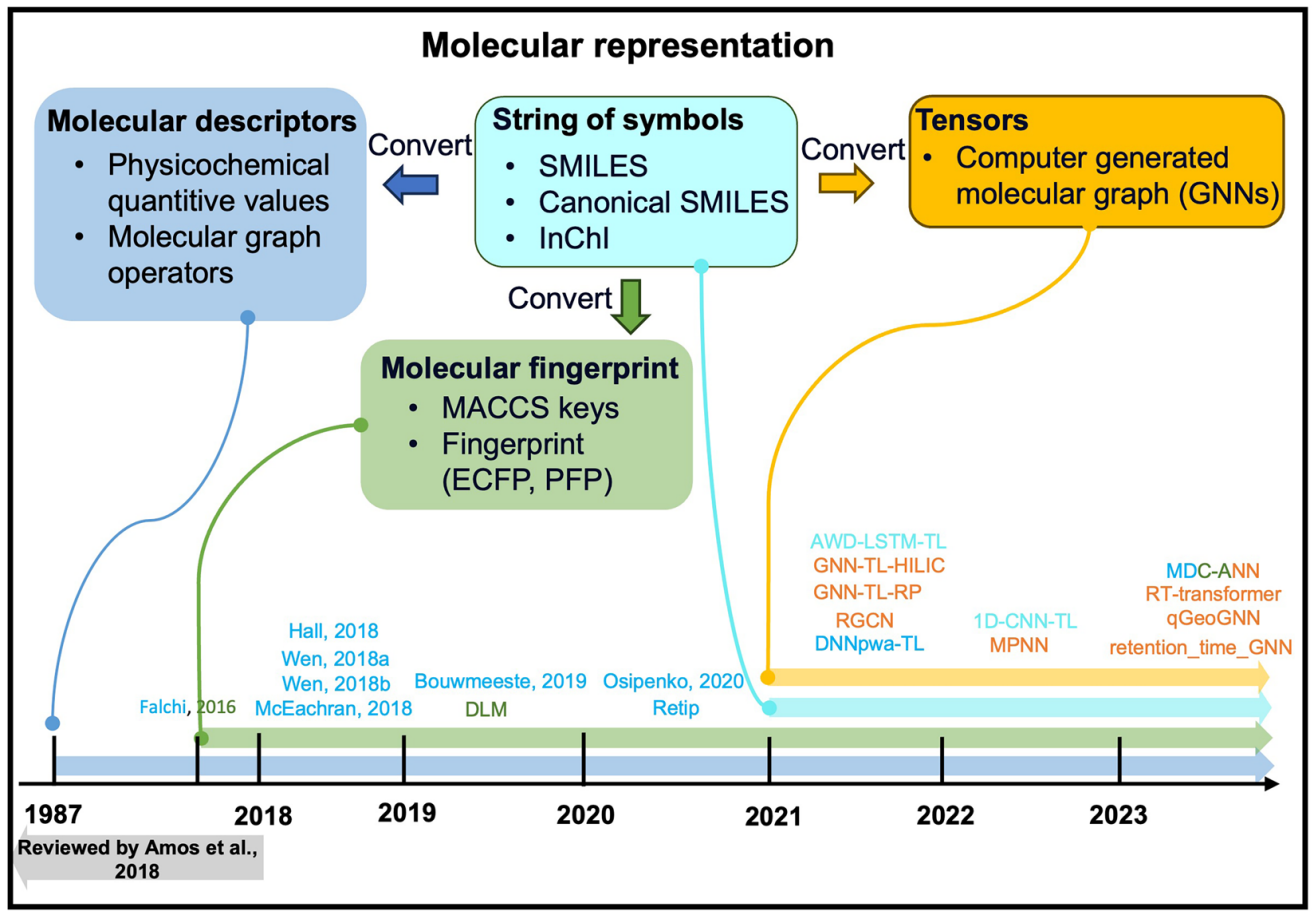
Molecular representations used in recent RT prediction models. MDC-ANN [36], RT-transformer [80], qGeoGNN [25], retention_time_GNN [37], 1D-CNN-TL [42], MPNN [70], AWD-LSTM-TL [56], GNN-TL-HILIC [46], GNN-TL-RP [45], RGCN [44], DNNpwa-TL [35], Osipenko [81], Retip [73], Bouwmeeste [34], DLM [23], Hall [82], Wen [83], Wen [84], McEachran [85], Falchi [86], and Amos et al. [40]

In terms of QSRR calculations for chromatography, the first step is to represent the molecular structure as interpretable data, such as vectors or numbers. Molecular representations, including molecular descriptors constituted by numerical values, topological fingerprint, such as MACCS keys, ECFP [23], text strings, such as SMILES [42], and graph neural network (GNN)-generated molecular graph [43–46] or their combinations [36, 37, 47], are used in RT prediction neural networks (Fig. 4).

### Molecular descriptors

Molecular descriptors in the form of numerical quantitative values of molecular physicochemical characteristics (solubility, boiling point, and lipophilicity) or geometry- and topological-related structural informatics features (adjacent matrix indices, distance matrix indices) are commonly used in QSRR [40], and over 6000 molecular descriptors can be generated for the mathematical representation of molecules using software, such as RDKit [38], RCDK [48], Mordret [49], or alvaDesc [50, 51]. Although the number of descriptors is normally reduced to tens or hundreds of levels through feature selection, thousands of descriptors have been fed into a robust model [47].

### Fingerprints

In addition to numerical descriptors, topology representations, such as MACCS keys and extended connectivity fingerprints (ECFP), represent the existence or nonexistence of molecular substructures in the form of numerical entry vectors and can complement molecular descriptors [36, 47]. In Domingo-Almenara et al.’s study, ECFP fingerprints were found to overperform compared to selected molecular descriptors as data input [23]. García et al. initially attempted to compare 5666 molecular descriptors only, 2214 molecular fingerprints (MACCS 166bit) only, and a combination of the two for molecular representation and found that there was not much difference in the performance of the three representations; therefore, only fingerprints were chosen to save computational resources [47]. Wang et al. explored the best combination of molecular descriptors, molecular fingerprints, and molecular graphs in 14 small datasets and found that, except for the three small datasets in which the combination of all is the best, the combination of molecular descriptors and molecular fingerprints is the best in the remaining 11 small datasets; they suggested that for independent small datasets, it is better to try multiple ways to decide the combination for best performance [36]. In Fedorova et al.'s study, 243 topological, constitutional, and electronic molecular descriptors were attempted individually and in combination, and the ECFP and physicochemical properties (PCP) molecular fingerprint representations and the canonical SMILES-transformed one-hot matrix representations were also input to the 1D-CNN model; it was found that molecular descriptors underperform, while SMILES-transformed one-hot matrices perform the best [42].

### Strings

SMILES and InChI strings can concisely represent atomic classes and their bonding modes, and can be converted into 2D-maps. One noteworthy difference is that SMILES are not unique, that is, the same molecule can be represented by multiple SMILES representations (canonical SMILES, Isomeric SMILES, etc.), whereas InChI has uniqueness and convertibility [52]. Because SMILES consists of English characters and symbols, it is widely used in pre-training tasks for natural language models to generate vector representation spaces for molecules, such as the pre-training models Smiles-Bert [53], Smiles transformer [54], and Chemformer [55], and is used in downstream RT prediction tasks [56].

### Molecular graphs

With the increasing demand for accurate 3D construction of molecules in fields, such as drug design, although molecular representations represented by strings and molecular fingerprints can represent the structure of molecules to a large extent owing to the lack of capturing the three-dimensional structure, attempts to utilize GNNs to approximate the construction of real-world molecules have been increasing in recent years [57]. Due to their flexibility and superiority in handling molecular graphs ranging from robust to precise spatial isomorphism levels, GNNs are widely used for various purposes, such as disease prediction and drug design, as highlighted by Zhang et al. [58]. Since their inception in 2005 [59], numerous variants of these networks have been extensively reviewed for molecular property predictions by Wieder et al. [60].

In molecular graph representation, a set of nodes and edges, ${\varvec{G}}=({\varvec{V}},\boldsymbol{ }{\varvec{E}})$ are used. Each node ${\varvec{v}}\boldsymbol{ }\in {\varvec{V}}$ is embedded with node feature vectors ${{\varvec{x}}}_{{\varvec{v}}}$, and each edge ${{\varvec{e}}}_{{\varvec{v}}{\varvec{w}}}\in {\varvec{E}}$ is embedded with edge feature vectors ${{\varvec{x}}}_{{\varvec{v}}{\varvec{w}}}^{{\varvec{e}}}$ that represent the connection between node ${\varvec{v}}$ and node ${\varvec{w}}$ [60]. Typically, the initial node representation ${{\varvec{h}}}_{{\varvec{v}}}^{0}$ is derived from the node feature vectors [61, 62]. Common types of node feature vectors include atom symbols, number of heavy atom neighbors, valence, aromaticity, etc. During the iteration, the node representation is updated by aggregating its neighbors' representations (see Eq. 3) and combining the aggregated representation with the node's previous feature vector (see Eq. 4). The graph representation is obtained using a permutation-invariant readout function, such as summation or maximization, which utilizes the final layer of the node representations (Eq. 5). The aggregation, combination, and readout methods differ across various GNN architectures [63, 64].

3 $$\varvec{a_{v}^{{\left( {l + 1} \right)}} = f_{Aggregate}^{{\left( {l + 1} \right)}} \left( {h_{v} ,h_{w}^{l} ,e_{vw} :w \in \left. {N_{\left( v \right)} } \right\}} \right)}$$

Aggregation of neighbor vectors

4 $$\varvec{h_{v}^{{\left( {l + 1} \right)}} = f_{Combine}^{{\left( {l + 1} \right)}} \left( {a_{v}^{{\left( {l + 1} \right)}} ,h_{v}^{l} } \right)}$$

Combination of $a_{v}^{{\left( {l + 1} \right)}}$ and previous feature vector

5 $$\varvec{h_{G} = f_{Readout} \left( {\left\{ {h_{v}^{L} :v \in G} \right\}} \right)}$$

Readout graph representation

$$l:\,layer\,L:\,last\,layer\,v:\,node\,v\,h:feature\,vector$$

$$N_{v} :\,neighbors\,of\,node\,v\,G:\,graph\,level\,representation$$

Yang et al. constructed a GNN model to extract subgraph features from a 2D molecular graph generated using an InChI identifier for six iterations. The subgraph vector is updated by summing the previous node state with the neighboring vector, which is then aggregated by a neural network. Stacking the last node state of the final iterations yields global graph representations for the downstream RT prediction task [45]. In addition, GNNs constructed in other fields have been successively applied to molecular representation in QSRR studies, three of which are introduced here:

#### (1) Graph convolutional network (GCN)

Kensert et al. employed the GNN variant GCN [61] and relational graph convolution network (RGCN) [65] models for the convolution of molecular graphs [44]. The atomic features, bonding features, and adjacency matrix were generated using SMILES with RDKit. In the GCN model, there are multiple graph convolutional layers (tuned as hyperparameters between 3 and 5 layers), and the radius of the neighborhood aggregation increases by one for each deeper layer. The inputs in each layer were the multiplication of normalized adjacency matrix ${\varvec{A}}$ (${\varvec{N}}\times {\varvec{N}}$, ${\varvec{N}}$ is the total number of atoms), feature matrix ${\varvec{H}}$ (${\varvec{N}}\boldsymbol{ }\times {\varvec{F}}$, ${\varvec{F}}$ is the feature dimension, ${\varvec{F}}$ in the first layer constituted by the 20 atomic features computed by RDKit, and the subsequent new features were the fusion of its own features and those of its neighbors), and weight matrix ${\varvec{W}}$ (${\varvec{F}}\times {{\varvec{F}}}^{\boldsymbol{^{\prime}}}$, ${{\varvec{F}}}^{\boldsymbol{^{\prime}}}$ is the number of neurons in the next layer), and the output was a ${\varvec{N}}\times {{\varvec{F}}}^{\boldsymbol{^{\prime}}}$ feature matrix, with a non-linear transformation function ${\varvec{\sigma}}$ (rectified linear unit function in Kensert et al*.*’s paper) as shown in Eq. 6:

6 $$\varvec{H^{{\left( {l + 1} \right)}} = \sigma \left( {\tilde{A}H^{\left( l \right)} W^{\left( 1 \right)} + H^{\left( l \right)} W^{\left( 0 \right)} } \right)}$$

The first part in parentheses in Eq. 6 contains the information of self and neighbor aggregation, whereas the latter part represents the information of the self-loop, which is linearly transformed with two weight matrices. After multilayer updating and average pooling, the tensor representation of the molecule was obtained and fed into the downstream fully connected layer for RT prediction. Its RGCN, on the other hand, introduces a more complex representation of the adjacency matrix ${\varvec{E}}$ (${\varvec{R}}\boldsymbol{ }\times {\varvec{N}}\boldsymbol{ }\times {\varvec{N}}$) with an extra dimension considering relations ${\varvec{R}}$; The update of the feature matrix is shown in Eq. 7:

7 $$\varvec{H^{{\left( {l + 1} \right)}} = \sigma \left( {H^{\left( l \right)} W_{0}^{\left( l \right)} + \mathop \sum \limits_{r = 0}^{R} \widetilde{{E_{r} }}H^{\left( l \right)} W_{r}^{\left( l \right)} } \right)}$$

In Eq. 7, ${\varvec{R}}$ stands for bond features, and ${\varvec{r}}$ represents each relational entity, such as single bond type, double bond type, etc. Consequently, the latter part of the formula in parentheses represents the cumulative aggregation of features across various bond features [44].

#### (2) Message-passing neural network (MPNN)

In 2017, Gilmer et al*.* proposed a framework called MPNN by unifying nine previous works and defined the MPNN as the message-passing phase (message functions, Eq. 8; vertex update functions, Eq. 9) and readout phase (Eq. 10). ${\varvec{N}}\left({\varvec{v}}\right)$ is the set of node ${\varvec{v}}$ neighbors; ${{\varvec{h}}}_{{\varvec{v}}}^{{\varvec{l}}},{{\varvec{h}}}_{{\varvec{w}}}^{{\varvec{l}}},{{\varvec{e}}}_{{\varvec{v}}{\varvec{w}}}$ denote the hidden states of node${\varvec{v}}$, node${\varvec{w}}$, and the edge feature between ${\varvec{v}}$ and${\varvec{w}}$; ${{\varvec{M}}}^{{\varvec{l}}}$ denotes the message function, and the sum of its outputs represents messages ${{\varvec{m}}}_{{\varvec{v}}}^{{\varvec{l}}+1}$ passing to node ${\varvec{v}}$ from its neighbors. The vertex state is updated by applying ${{\varvec{U}}}^{{\varvec{l}}}$ which denotes the vertex update function on node ${\varvec{v}}$’s hidden state and passing messages from its neighbors. The readout function ${\varvec{R}}$ operates on a set of nodes in the graph in the final layer ${\varvec{L}}$ to obtain graph-level output [66].

8 $$\varvec{m_{v}^{l + 1} = \mathop \sum \limits_{w \in N\left( v \right)} M^{l} \left( {h_{v}^{l} ,h_{w}^{l} ,e_{vw} } \right)}$$

Message from neighbors

9 $$\varvec{h_{v}^{l + 1} = U^{l} \left( {h_{v}^{l} ,m_{v}^{l + 1} } \right)}$$

vertex update

10 $$\varvec{\hat{y} = R\left( {\left\{ {h_{v}^{L} \,\left| {v \in G} \right.} \right\}} \right)}$$

readout.

In Gilmer et al*.*’s MPNN variants, the application of an edge network as a message function (Eq. 11), GRU as a vertex update function (Eq. 12), and the set2set model [67] as the readout function achieved over-performance in dealing with molecular property prediction tasks in the field of quantum chemistry [66].

11 $$\varvec{M^{l} \left( {h_{v}^{l} ,h_{w}^{l} ,e_{vw} } \right) = NN\left( {e_{vw} } \right)h_{w}^{l}}$$

12 $$\varvec{U^{l} \left( {h_{v}^{l} ,m_{v}^{l + 1} } \right) = GRU\left( {h_{v}^{l} ,m_{v}^{l + 1} } \right)}$$

The framework of Gilmer et al*.*’s MPNN variant was implemented in the DeepChem library [68], providing an easy-to-use method that was used by Xing et al. to generate vector representations for 398 authentic compounds with four-step message-passing and four-step set-to-set model computations for readout [43]. Osipenko et al. followed the Keras implementation of the MPNN [69], which utilizes a transformer encoder and average pooling instead of the set-to-set layer in the readout phase of Gilmer et al*.*’s MPNN [70].

#### (3) Graph isomorphism network (GIN)

GIN can distinguish graph structures and capture local structure information by utilizing a more powerful aggregate strategy. As a practical example, in the original paper published in 2018 [63], the node representation can be updated according to Eq. 13:

13 $$\varvec{h_{v}^{\left( l \right)} = MLP^{\left( l \right)} \left( {\left( {1 + \varepsilon^{\left( l \right)} } \right) \cdot h_{v}^{{\left( {l - 1} \right)}} + \mathop \sum \limits_{w \in N\left( v \right)} h_{w}^{{\left( {l - 1} \right)}} } \right)}$$

The parameter ${\varvec{\epsilon}}$ serves as a learnable scaler or adjustment factor, fine-tuning the balance between a node's own features and those of its neighbors. Moreover, a multilayer perceptron (MLP) enhances the ability to extract features more effectively than simpler functions, such as summation, averaging, or max pooling. Additionally, they proposed a readout function as a concatenation of each iteration by summing all node representations for graph-level representations [63]. In Kwon et al.’s research, their RT prediction model was constructed using five layers of the revised GIN architecture. The ${\varvec{\epsilon}}$ is set to zero, and neighbor messages are represented by the summation of node feature vectors and its edge feature vectors with activation of rectiled linear unit (ReLU) function. In addition, the readout function uses average pooling, which differs from that of the original GIN architecture [37]. Based on GIN, a geometry-enhanced molecular representation learning method (GEM) is proposed to enhance the capture of molecular geometry knowledge [62]. In addition to the atom-bond graph represented by Eq. 8, the edge representation ${{\varvec{e}}}_{{\varvec{v}}{\varvec{w}}}^{{\varvec{l}}}$ was learned using an additional GNN that embedded the bond-angle features. For a precise consideration of the 3-D molecular structure details, Xu et al. constructed a quantile geometry-enhanced graph neural network (QGeoGNN) for chiral molecular separations, considering their isomorphism based on GEM [25].

In all the aforementioned GNNs, the selection of atomic features was different and appeared to be subjective. Pocha et al*.* evaluated the effect of atomic feature selection on the GNN performance for molecular property prediction tasks [71]. It was found that more atomic features tended to perform better; however, removing aromaticity, inclusion in a ring, and formal charge features, or adding heavy neighbors and hydrogen features could improve model performance.

## Application of neural networks in QSRR

Over a long period of time, the modeling method in QSRR mainly integrated ML linear or non-linear regression algorithms and was usually performed on small in-house or public datasets at the level of hundreds or thousands of compounds [27, 29, 72]. Bouwmeester et al. evaluated the prediction performance of seven ML algorithms: Bayesian ridge regression (BRR), least absolute shrinkage and selection operator (LASSO), deep neural networks (DNNs), adaptive boosting (AB), gradient boosting (GB), random forest (RF), and supported vector regression (SVR), and their relationship with dataset size on 36 small datasets. It found significant variations among different small datasets, and while the GB algorithm was relatively more likely to have a performance advantage, no single ML algorithm could perform optimally on all performance sets [34].

Following the release of the SMRT dataset in 2019, there has been a surge in research focusing on training deep-learning models on this extensive dataset. The explored modeling architectures include deep neural network (DNN) (DNNpwa-TL, CMM-RT) [35, 47], convolutional neural network (CNN) (1D CNN-TL) [42], recurrent neural network (RNN) (AWD-LSTM) [56], transformer (TransformerXL) [56], adaptive neural network (ANN) (MDC-ANN) [36] and GNN [25, 37] (Table 6). Typically, these methodologies involve projecting information from the large dataset onto small datasets containing a select few benchmark chemicals, referred to as ''anchor compounds'' [23], or employing transfer learning techniques on smaller datasets [35–37, 44, 70].

**Table 6:** Retention time prediction models established in the last 5 years (2019–2023)

Name	Molecular representation	Train set	Test set	Model structure	Comparison method	Refs.
retention_time_GNN	Molecular graph trained by GNN	SMRT	24 SD including PredRet, MoNA, and in-house datasets	GIN + Broyden–Fletcher–Goldfarb–Shanno (L-BFGS) optimizer + TL	MDC-ANN, DNNpwa-TL, GNN-RT-TL, GCN, RGCN, and 1D-CNN	[37]
QGeoGNN	Molecular graph integrating descriptors and column propert	CMRT (25847 data collected from 644 reports)	Train set split (5% validation; 5% test)	GeoGNN + quantile loss learning	LGB, XGB, Artificial neural network, and GNN	[25]
RT-transformer	Molecular graph with 34 features per node and 5 features per bond generated by RDKit	SMRT	41 PredRet SDs	GAT + 1D-transformer	GNN-RT, 1D-CNN; Blender, CPORT, and MPNN	[80]
Multi-data combination of compounds and adaptive neural network (MDC-ANN)	(1) Molecular graph with 25 features (referred [45]; (2) molecular fingerprint from MACCS calculated by RDKit; (3) molecular descriptors from SMILEs calculated by Mordred and remain 1156 features by pre-processing	SMRT train, 1511 in-house standards for fine-tuning;	14 small datasets and 807 in-house standards	DNN model based on different combinations of molecular presentations	DNNpwa-TL, GNN-RT-TL [45], GCN, RGCN, 1D-CNN, RF, GB, and LASSO and XGBoost, MDC-CNN	[36]
Multi-target QSRR models (mt-QSRR)	225 constitutional, topological, and geometrical descriptors as numerical characteristics molecular descriptors by RDKit	Seven standards at five different pH conditions (2.7, 3.5, 5.0, 6.5, and 8.0)	Train set split	RF	–	[87]
CMM-RT	5666 MD and 2214 fingerprints (MACCS166) generated by alvaDesc software	SMRT	PredRet (FEM_long, FEM_obitrap_plasma, LIFE_old, RIKEN)	DNN	SVM, XGB, LGB, CatBoost, and a blending approach	[47]
Message-passing neural networks (MPNN)	Molecular graph with 5 features per node and 2 features per bond	SMRT	PredRet (FEM_long, LIFE_new, LIFE_old, Eawag_XBridgeC18) and RIKEN Retip SD	MPNN	1D-CNN and GNN [45]	[70]
1D-CNN; 1D-CNN-TL	(1) Topological, constitutional, and electronic molecular descriptors test separated and combined (243 in total) by RCDK (2) ECFP/PCP fingerprints by RCDK; (2) SMILES to one-hot matrice	SMRT (for 1D-CNN training)	5 SD (RIKEN_Retip, MassBank1, MetaboBASE, LIFE_old, LIFE_new) for TL (10 validation)	1D CNN + TL	DLM [23] XGB [81], and GNN [45]	[42]
	70–92 MDs by RDKit	26–350 STDs	SMRT	BRidgeR, XGBR and SVR	–	[88]
MultiConditionRT	153 MDs	78 STDs	151 (internal) & 324 [91]	RF	–	[89]
HighResNPS	4 MDs + one-hot encoding	707 STDs train, 190 STDs optimization, 191 STDs validation	193 STDs	MLP	–	[90]
GNN-RT-TL	Molecular Graph generate from InChI by RDKit, and extract subgraph by GNN	SMRT (for GNN training)	11 SDs from MoNA and PredRet (TL)	GNNs + TL	Multichannel-CNN (MC-CNN), single channel-CNN (SC-CNN), BRR [34], RFs [34], DLM [23]	[45]
GNN-TL	Molecular graph with 16 features per node and 4 features per bond	In silico HILIC RT dataset with about 306 K molecules (for GNNs training)	880 compounds (Fiehn HILIC) for TL	GNNs + TL	Retip (XGB, BRNN, RF, LGB, Keras DNN)	[46]
RGCN, GCN	Molecular graph with 20features per node and 5 features per bond for RGCN; 20 features per node for GCN generated by RDKit	SMRT train,validation, and test splits follow [45]; RIKEN and Fiehn HILIC follow [73]	External test set in[73]; Train set split	GCN and RGCN	MLP with ECFP, and RF, SVM, GB, AB with descriptors, GNN-RT, and Keras	[44]
DNNpwa-TL	1470 MDs calculated by Mordred	SMRT pre-training by AE-wmi	17 SDs for TL (1055 TL + 133 test)	DNNs + TL	CALLC, PredRet, RF, GB, LASSO, DNN, and GNN-RT [45]	[35]
mixed-mode-MPNN	SMILES	398 STDs	Train set split (20%)	MPNN (from DeepChem library [68])	Linear regression; RF	[43]
	4 non-canonical + 1 canonical SMILES	ChEMBL 1 million molecules (pre-training)	4 SDs (Eawag_XBridgeC18, Beck, Stravs, and FEM_long) for TL	FastAI AWD-LSTM/ TransformerXL + TL (median fine-tuning on SMRT, second fine-tuning on SDs than regression fine-tuning generates RT)	AB, BRR, RF, SVR, and GB	[56]
Retip	286 MDs	981 (HILIC,Fiehn, MoNA) & 852 (RP-LC,PlaSMA) STDs	143 metabolites (human blood plasma MS/MS data) as external set	XGB, BRNN, RF, LGB, Keras DNN (3 dense layer + 3 dropout layer)	XGB, BRNN, RF, LGB, Keras DNN (3 dense layer + 3 dropout layer)	[73]
DLM; SMRT	ECFP fingerprint	80,038 STDs	PredRet SDs	Keras deep-learning regression model + projection by robust polynomial regression to SDs	RF	[23]
	151 MDs	6,759 STDs from 36 public small datasets	Train set split	BRidgeR, LASSO, DNNs, AB, GB, RF, and SVR	BRidgeR, LASSO, DNNs, AB, GB, RF, and SVR	[34]

*MDs* molecular descriptors, *SDs* small datasets, *STD* standard chemical

Although the model architectures varied, the findings of these studies have the following points in common: (1) neural network architectures generally outperform traditional regression algorithms, such as partial least squares regression (PLS), RF, SVM, LASSO, and GB [35, 44]; (2) transfer learning generally achieves better performance than building models from scratch on small datasets. Transfer learning of 1D-CNN [42], AWD-LSTM [56], and TransformerXL [56] models achieved better performance than learning from scratch on most of the small datasets. (3) Although the models and the small datasets used for comparison vary, no single model has yet achieved absolute superiority across all the small datasets tested, as reported in published articles [36, 37]. (4) Model performance is affected by the molecular similarity between testing and training datasets; the more similar they are, the better the performance. In Xu et al.'s study, molecules in the test set with more than 90% similarity to the training set resulted in satisfactory performance; however, the prediction accuracy significantly decreased as the molecular similarity decreased [25], a finding similar to that of Domingo-Almenara et al*.*'s observation [23]. Although the performance degradation on the test set is technically attributed to a lack of model generalization, considering the vast latent space of chemical structures, it may require great efforts or skillful strategies to overcome this challenge.

Current study primarily employed a loss function based on the mean square error (MSE) between the predicted and labeled RTs, focusing on accuracy. However, in actual applications, even within the same chromatographic system, the RT of compounds can vary within a small range owing to unavoidable errors. Osipenko et al*.*'s study simulated this type of variability by adding Gaussian noise with a standard deviation of five seconds to real data labels [56]. Xu et al. used quantile learning to account for the uncertainty in RTs by incorporating quantile loss in the loss function, which assessed the probability of separation and improved fault tolerance [25].

## Practicality of metabolite annotation

Considering that the RT prediction model is ultimately applied to practical metabolite annotation, the following two points require special attention and discussion.

### (1) Difficulty in the implementation of in-house datasets

This includes the number of known compounds required for application in laboratory LC systems. Although more training instances generally lead to better training outcomes, the availability of known training instances such as standards is limited in the laboratory. Thus, achieving high prediction accuracy with fewer training instances is of practical significance. The three current implementation methods, training from scratch, transfer learning, and projection functions, differ in their training instance requirements (Table 7).

**Table 7:** Number of known compounds required for application in laboratory systems

	Recommend instances	Refs.
Training in-house RT libraries		
Retip	300	[73]
Bouwmeester et al.	40–100	[34]
Transfer learning		
Ju et al.	73–665 (90% in datasets)	[35]
Yang et al.	100–200	[46]
Projection function		
Domingo-Almenara et al.	50 anchor-compounds	[23]
García et al.	10	[47]

Training in-house RT libraries to build models is a common method, and more than 300 training instances are recommended for Retip model building [73]. In a study by Bouwmeester et al. training from scratch on PredRet's five small datasets with seven ML methods required at least 40 instances (the best method in three datasets achieved a mean absolute error (MAE) between 100 and 120 s) and providing 100 instances showed significant improvement (the best method in three datasets achieved an MAE of less than 100 s) [34].

Transfer learning requires fine-tuning small datasets, and according to currently published information, it usually involves selecting small datasets with over hundred known compounds. Ju et al. conducted transfer learning on 17 small datasets ranging from 73 to 665 instances using 90% of the training instances in each dataset for fine-tuning [35]. Yang et al. indicated that a pre-trained GNN model on a 306 K dataset, when transferred to small HILIC datasets, required 150 training instances to achieve higher accuracy (MAE of approximately 30 s), suggesting that approximately 100–200 training instances are required in actual applications [46].

After training the models on large datasets, the projection function mapped the predicted RT to a specific chromatographic method with only a few identified molecules. For example, Domingo-Almenara et al. used robust polynomial regression for projection, achieving the objective of 70% correct molecular identity ranked among the top three candidates with only 50 anchor-compound examples from PredRet small datasets [23]. García et al. projected, using a Bayesian meta-learning approach, achieving 68% correct molecular identity in the top three candidates filtered by exact mass with only ten known compounds on four PredRet small datasets [47]. However, the performance of the projection method may not be as good as that of transfer learning. Kwon et al. made a commendable effort to compare four different model constructions: (1) learning from scratch, (2) transfer learning using feature extraction with two types of optimizers (similar to Yang et al*.* [45, 46] and Osipenko et al*.* [70] methods), (3) transfer learning by fine-tuning with two types of optimizers, and (4) polynomial regression projection (similar to Domingo-Almenara et al. [23] method) on 24 small datasets. The evaluation results ranked the prediction errors in the following ascending order: transfer learning by fine-tuning, transfer learning using feature extraction, learning from scratch, and polynomial regression projection [37]. In our experience, García et al.'s requirement for ten known compounds [47] aligns more closely with actual application scenarios in laboratories, and constructing an in-house library with hundreds of compounds under the same chromatography system conditions is very challenging. Therefore, meeting the practical needs of both low requirements for the number of known compounds in in-house libraries and achieving high prediction accuracy remains an ongoing challenge.

### (2) Ability to eliminate incorrect options

Enhancing the accuracy of metabolite annotation is an important application of RT prediction. Therefore, in addition to evaluating errors, such as MRE and MAE, it is crucial to assess the efficiency of eliminating incorrect metabolite annotation options. Domingo-Almenara et al. evaluated the capability of a DL model trained on SMRT to select correct options on small datasets [23]. By predicting the RTs for 6832 compounds with Kyoto encyclopedia of genes and genomes (KEGG) entries [74] and mapping these RTs to small datasets via projection, candidates were ranked by errors between the projected and observed RTs. It found that 70% of the correct options were among the top three candidates [23]. Bonini et al. demonstrated how retip-assisted MS-DIAL [75] eliminated false-positive examples of mouse plasma metabolomics data, where the predicted RTs were beyond the one-minute observation RT limit [73]. The study by Yang et al. on the HILIC system detailed the changes in the ranking of correct options before and after GNN-TL help MS-FINDER [76] annotate 100 metabolites from three small datasets. The results indicated that, except for one false negative and one compound whose ranking decreased, the rankings of all other correct options either increased or remain unchanged [46]. Notably, RT is a significant reference value for distinguishing structural isomers. Therefore, the differentiation of structural isomers warrants further exploration.

## Development of RT projection methodology for metabolite annotation

The projection method aims to design a system that enables the comparison of RTs from different laboratory systems. PredRet [32] provides an R package and a user-friendly website interface for predicting RTs across shared experimental systems. By mapping an LC system to an existing one based on the overlap of annotations, the RTs of metabolites annotated in the referenced system can be predicted and warnings for outlier prediction can be issued. Improved accuracy was achieved by calibrating the RTs using a regression algorithm across different LC setups, resulting in a higher accuracy performance than the SVR-based ML model reported by Aicheler et al. [33, 77]. To evaluate the RT prediction performance of PredRet for plant food bioactive compounds, 1583 experimental analytes (467 metabolites) from 24 LC systems were tested, obtaining acceptable median prediction errors within the 0.3–1.8% range. It exhibited a clear distinction between two pairs of structural isomers (veratric acid, homovanillic acid, dihydrocaffeic acid and kaempfeol, luteolin, fisetin), highlighting its practical application [78].

## Conclusions

In this review, we acknowledged two major challenges in directly comparing accuracy or metric values without reproducing all models on a uniform benchmark for the testing sets and the models used for comparison were varied (refer to Table 6 under 'Test Set' and 'Comparison Method'). Consequently, we refrained from focusing on reporting evaluation metrics such as MAE, MRE, or median absolute error (MedAE). This highlight the urgent need for a standardized benchmark, such as MoleculeNet [79], which was designed for molecular property prediction in molecular ML and includes a compilation of public datasets and evaluation metrics. Considering the widespread application of deep-learning models for RT prediction, extensive training datasets are required. Availability of large datasets, such as those on SMRT and RepoRT will accelerate the development of DL-based models. We anticipate the release of additional training resources in the future. To enhance practicality, evaluation of RT prediction models should not only focus on accuracy but also on the capacity to eliminate false candidates, regardless of assistance by MS annotation. Ability to discriminate between structural isomers, especially functional group isomerism and positional isomerism, is a key application of RT for metabolite identification. Therefore, this aspect should be further evaluated as structural isomer distinction poses a challenge to metabolite annotation.

As discussed in section ''Discussion about representations in small molecular RT datasets'', inconsistent molecular representations may lead to fundamental errors in studies depending on the identifiers used, as illustrated in Fig. 3D. As additional information, we recommend that CAS registry numbers be provided for studies using commercial standards wherever possible, to prevent significant misunderstandings. In such cases, further manual efforts may be required for checking and revising the data.

In relation to the compound structure, there can be a distinction between the 'injected compound structure' and the 'in-solution structure' due to the presence of an additive salt. If this salt is irrelevant to the RT, the InChI, as referenced by the CAS number, may need to be 'cleaned up' or 'standardized' to accurately reflect the structure of the compound being injected.

For practical applications, LC systems are frequently adjusted to separate various sample types, typically running only a limited number of standards (e.g., 5–20) alongside testing samples under new conditions. This makes it challenging to calibrate in-house models for specific LC systems due to the extensive need for standards. Thus, achieving a balance between model prediction accuracy and the required quantity of in-house compounds by leveraging both RT and *m/z* represents a valuable goal. Furthermore, the development of intuitive documentation and APIs, integrated with MS annotation tools like MS-DIAL [75] or MS-FINDER [76], will enhance researcher usability. As metabolic annotation progresses with technological advances, we anticipate that software-supported metabolite annotation will increasingly assist laboratory scientists in the future.

## Supplementary Information

Supplementary material 1.

## Data Availability

No datasets were generated or analysed during the current study.

## Abbreviations

AB: Adaptive boosting
ANN: Adaptive neural network
BRidgeR: Bayesian ridge regression
BRNN: Bayesian-regularized neural network
CAS: Chemical abstracts service
CNN: Convolutional neural network
DL: Deep learning
DNN: Deep neural network
ECFP: Extended-connectivity fingerprint
GB: Gradient boosting
GC: Gas-chromatography
GCN: Graph convolutional network
GNN: Graph neural network
HILIC: Hydrophilic interaction liquid chromatography
InChI: IUPAC international chemical identifier
InChIKey: International chemical identifier key
KEGG: Kyoto encyclopedia of genes and genomes
LASSO: Least absolute shrinkage and selection operator
LC: Liquid chromatography
LGB: Light gradient-boosting machine
MAE: Mean absolute error
MDC-ANN: Multi-data combinations and adaptive neural network
MedAE: Median absolute error
ML: Machine learning
MLP: Multilayer perceptron
MRE: Mean relative error
MS: Mass spectrometry
RF: Random forest
RGCN: Relational graph convolutional network
RNN: Recurrent neural network
RT: Retention time
SMILES: Simplified molecular-input line-entry system
SVR: Supported vector regression
TL: Transfer learning
XGB: XGBoost
XGBR: Extreme gradient boosting regression

## Code availability

Source code for analyzing current states of RT datasets: https://github.com/LiuLime/PredRT_review_2024.git.

## References

[1] Jordan MI, Mitchell TM (2015) Machine learning: trends, perspectives, and prospects. Science 349(6245):255–260 26185243 10.1126/science.aaa8415

[2] Dührkop K, Shen H, Meusel M, Rousu J, Böcker S (2015) Searching molecular structure databases with tandem mass spectra using CSI: FingerID. Proc Natl Acad Sci 112(41):12580–12585 26392543 10.1073/pnas.1509788112 PMC4611636

[3] Dührkop K, Fleischauer M, Ludwig M, Aksenov AA, Melnik AV, Meusel M, Dorrestein PC, Rousu J, Böcker S (2019) SIRIUS 4: a rapid tool for turning tandem mass spectra into metabolite structure information. Nat Methods 16(4):299–302 30886413 10.1038/s41592-019-0344-8

[4] Wei JN, Belanger D, Adams RP, Sculley D (2019) Rapid prediction of electron–ionization mass spectrometry using neural networks. ACS Cent Sci 5(4):700–708 31041390 10.1021/acscentsci.9b00085 PMC6487538

[5] Wang F, Liigand J, Tian S, Arndt D, Greiner R, Wishart DS (2021) CFM-ID 4.0: more accurate ESI-MS/MS spectral prediction and compound identification. Anal Chem 93(34):11692–11700 34403256 10.1021/acs.analchem.1c01465 PMC9064193

[6] MoNA-MassBank of North America. https://mona.fiehnlab.ucdavis.edu/. Accessed 11 Nov 2023.

[7] Stravs MA, Dührkop K, Böcker S, Zamboni N (2022) MSNovelist: de novo structure generation from mass spectra. Nat Methods 19(7):865–870 35637304 10.1038/s41592-022-01486-3 PMC9262714

[8] Shrivastava AD, Swainston N, Samanta S, Roberts I, Wright Muelas M, Kell DB (2021) MassGenie: a transformer-based deep learning method for identifying small molecules from their mass spectra. Biomolecules 11(12):1793 34944436 10.3390/biom11121793 PMC8699281

[9] Nicoud R-M (2015) Chromatographic processes. Cambridge University Press, Cambridge

[10] Wishart DS, Guo A, Oler E, Wang F, Anjum A, Peters H, Dizon R, Sayeeda Z, Tian S, Lee BL (2022) HMDB 5.0: the human metabolome database for 2022. Nucleic Acids Res 50(D1):D622–D631 34986597 10.1093/nar/gkab1062 PMC8728138

[11] Wang M, Carver JJ, Phelan VV, Sanchez LM, Garg N, Peng Y, Nguyen DD, Watrous J, Kapono CA, Luzzatto-Knaan T (2016) Sharing and community curation of mass spectrometry data with global natural products social molecular networking. Nat Biotechnol 34(8):828–837 27504778 10.1038/nbt.3597 PMC5321674

[12] Sawada Y, Nakabayashi R, Yamada Y, Suzuki M, Sato M, Sakata A, Akiyama K, Sakurai T, Matsuda F, Aoki T (2012) RIKEN tandem mass spectral database (ReSpect) for phytochemicals: a plant-specific MS/MS-based data resource and database. Phytochemistry 82:38–45 22867903 10.1016/j.phytochem.2012.07.007

[13] Horai H, Arita M, Kanaya S, Nihei Y, Ikeda T, Suwa K, Ojima Y, Tanaka K, Tanaka S, Aoshima K (2010) MassBank: a public repository for sharing mass spectral data for life sciences. J Mass Spectrom 45(7):703–714 20623627 10.1002/jms.1777

[14] Smith CA, O’Maille G, Want EJ, Qin C, Trauger SA, Brandon TR, Custodio DE, Abagyan R, Siuzdak G (2005) METLIN: a metabolite mass spectral database. Ther Drug Monit 27(6):747–751 16404815 10.1097/01.ftd.0000179845.53213.39

[15] AIST. Spectral Database for Organic Compounds, AIST. https://sdbs.db.aist.go.jp/sdbs/cgi-bin/direct_frame_top.cgi.

[16] NIST Mass Spectral Libraries, 2023 Edition with Search Program Data Version: NIST23. https://www.nist.gov/srd/nist-standard-reference-database-1a.

[17] METLIN Gen2. https://massconsortium.com/.

[18] mzCloud™ spectral library. https://www.mzcloud.org/.

[19] Wiley Registry of Tandem Mass Spectral Data, MS for ID. https://www.wiley.com/en-gb/Wiley+Registry+of+Tandem+Mass+Spectral+Data%2C+MS+for+ID-p-9781118037447. 10.1002/jms.3185 23584943

[20] Vinaixa M, Schymanski EL, Neumann S, Navarro M, Salek RM, Yanes O (2016) Mass spectral databases for LC/MS-and GC/MS-based metabolomics: state of the field and future prospects. TrAC, Trends Anal Chem 78:23–35

[21] Yurekten O, Payne T, Tejera N, Amaladoss FX, Martin C, Williams M, O’Donovan C (2023) MetaboLights: open data repository for metabolomics. Nucleic Acids Res. 10.1093/nar/gkad1045 10.1093/nar/gkad1045 PMC10767962 37971328

[22] Sud M, Fahy E, Cotter D, Azam K, Vadivelu I, Burant C, Edison A, Fiehn O, Higashi R, Nair KS (2016) Metabolomics Workbench: an international repository for metabolomics data and metadata, metabolite standards, protocols, tutorials and training, and analysis tools. Nucleic Acids Res 44(D1):D463–D470 26467476 10.1093/nar/gkv1042 PMC4702780

[23] Domingo-Almenara X, Guijas C, Billings E, Montenegro-Burke JR, Uritboonthai W, Aisporna AE, Chen E, Benton HP, Siuzdak G (2019) The METLIN small molecule dataset for machine learning-based retention time prediction. Nat Commun 10(1):5811 31862874 10.1038/s41467-019-13680-7 PMC6925099

[24] Kretschmer F, Harrieder E-M, Hoffmann MA, Böcker S, Witting M (2024) RepoRT: a comprehensive repository for small molecule retention times. Nat Methods. 10.1038/s41592-023-02143-z 38191934 10.1038/s41592-023-02143-z

[25] Xu H, Lin J, Zhang D, Mo F (2023) Retention time prediction for chromatographic enantioseparation by quantile geometry-enhanced graph neural network. Nat Commun 14(1):3095 37248214 10.1038/s41467-023-38853-3 PMC10227049

[26] Eugster PJ, Boccard J, Debrus B, Bréant L, Wolfender J-L, Martel S, Carrupt P-A (2014) Retention time prediction for dereplication of natural products (CxHyOz) in LC–MS metabolite profiling. Phytochemistry 108:196–207 25457501 10.1016/j.phytochem.2014.10.005

[27] Broeckling CD, Ganna A, Layer M, Brown K, Sutton B, Ingelsson E, Peers G, Prenni JE (2016) Enabling efficient and confident annotation of LC− MS metabolomics data through MS1 spectrum and time prediction. Anal Chem 88(18):9226–9234 27560453 10.1021/acs.analchem.6b02479

[28] Bruderer T, Varesio E, Hopfgartner G (2017) The use of LC predicted retention times to extend metabolites identification with SWATH data acquisition. J Chromatogr B 1071:3–10 10.1016/j.jchromb.2017.07.016 28780068

[29] Cao M, Fraser K, Huege J, Featonby T, Rasmussen S, Jones C (2015) Predicting retention time in hydrophilic interaction liquid chromatography mass spectrometry and its use for peak annotation in metabolomics. Metabolomics 11:696–706 25972771 10.1007/s11306-014-0727-x PMC4419193

[30] Arapitsas P, Speri G, Angeli A, Perenzoni D, Mattivi F (2014) The influence of storage on the “chemical age” of red wines. Metabolomics 10:816–832

[31] Stravs MA, Schymanski EL, Singer HP, Hollender J (2013) Automatic recalibration and processing of tandem mass spectra using formula annotation. J Mass Spectrom 48(1):89–99 23303751 10.1002/jms.3131

[32] Stanstrup J, Neumann S, Vrhovsek U (2015) PredRet: prediction of retention time by direct mapping between multiple chromatographic systems. Anal Chem 87(18):9421–9428 26289378 10.1021/acs.analchem.5b02287

[33] Bouwmeester R, Martens L, Degroeve S (2020) Generalized calibration across liquid chromatography setups for generic prediction of small-molecule retention times. Anal Chem 92(9):6571–6578 32281370 10.1021/acs.analchem.0c00233

[34] Bouwmeester R, Martens L, Degroeve S (2019) Comprehensive and empirical evaluation of machine learning algorithms for small molecule LC retention time prediction. Anal Chem 91(5):3694–3703 30702864 10.1021/acs.analchem.8b05820

[35] Ju R, Liu X, Zheng F, Lu X, Xu G, Lin X (2021) Deep neural network pretrained by weighted autoencoders and transfer learning for retention time prediction of small molecules. Anal Chem 93(47):15651–15658 34780148 10.1021/acs.analchem.1c03250

[36] Wang X, Zheng F, Sheng M, Xu G, Lin X (2023) Retention time prediction for small samples based on integrating molecular representations and adaptive network. J Chromatogr B 1217:123624 10.1016/j.jchromb.2023.123624 36780745

[37] Kwon Y, Kwon H, Han J, Kang M, Kim J-Y, Shin D, Choi Y-S, Kang S (2023) Retention time prediction through learning from a small training data set with a pretrained graph neural network. Anal Chem. 10.1021/acs.analchem.3c03177 37955847 10.1021/acs.analchem.3c03177

[38] RDKit. https://www.rdkit.org. Accessed 01 Dec 2023.

[39] ClassyFire Batch by Fiehn Lab. https://cfb.fiehnlab.ucdavis.edu/. Accessed 01 Dec 2023.

[40] Amos RI, Haddad PR, Szucs R, Dolan JW, Pohl CA (2018) Molecular modeling and prediction accuracy in quantitative structure-retention relationship calculations for chromatography. TrAC, Trends Anal Chem 105:352–359

[41] Haddad PR, Taraji M, Szücs R (2020) Prediction of analyte retention time in liquid chromatography. Anal Chem 93(1):228–256 33085452 10.1021/acs.analchem.0c04190

[42] Fedorova ES, Matyushin DD, Plyushchenko IV, Stavrianidi AN, Buryak AK (2022) Deep learning for retention time prediction in reversed-phase liquid chromatography. J Chromatogr A 1664:462792 34999303 10.1016/j.chroma.2021.462792

[43] Xing G, Sresht V, Sun Z, Shi Y, Clasquin MF (2021) Coupling mixed mode chromatography/ESI negative MS detection with message-passing neural network modeling for enhanced metabolome coverage and structural identification. Metabolites 11(11):772 34822429 10.3390/metabo11110772 PMC8620857

[44] Kensert A, Bouwmeester R, Efthymiadis K, Van Broeck P, Desmet G, Cabooter D (2021) Graph convolutional networks for improved prediction and interpretability of chromatographic retention data. Anal Chem 93(47):15633–15641 34780168 10.1021/acs.analchem.1c02988

[45] Yang Q, Ji H, Lu H, Zhang Z (2021) Prediction of liquid chromatographic retention time with graph neural networks to assist in small molecule identification. Anal Chem 93(4):2200–2206 33406817 10.1021/acs.analchem.0c04071

[46] Yang Q, Ji H, Fan X, Zhang Z, Lu H (2021) Retention time prediction in hydrophilic interaction liquid chromatography with graph neural network and transfer learning. J Chromatogr A 1656:462536 34563892 10.1016/j.chroma.2021.462536

[47] García CA, Gil-de-la-Fuente A, Barbas C, Otero A (2022) Probabilistic metabolite annotation using retention time prediction and meta-learned projections. J Cheminform 14(1):1–23 35672784 10.1186/s13321-022-00613-8 PMC9172150

[48] Guha R (2007) Chemical informatics functionality in R. J Stat Softw 18:1–16

[49] Moriwaki H, Tian Y-S, Kawashita N, Takagi T (2018) Mordred: a molecular descriptor calculator. J Cheminform 10(1):1–14 29411163 10.1186/s13321-018-0258-y PMC5801138

[50] Mauri A (2020) alvaDesc: a tool to calculate and analyze molecular descriptors and fingerprints. Ecotoxicol QSARs. 10.1007/978-1-0716-0150-1_32

[51] Mauri A, Bertola M (2022) Alvascience: a new software suite for the QSAR workflow applied to the blood–brain barrier permeability. Int J Mol Sci 23(21):12882 36361669 10.3390/ijms232112882 PMC9655980

[52] Elton DC, Boukouvalas Z, Fuge MD, Chung PW (2019) Deep learning for molecular design—a review of the state of the art. Mol Syst Des Eng 4(4):828–849

[53] Wang S, Guo Y, Wang Y, Sun H, Huang J (2019) Smiles-bert: large scale unsupervised pre-training for molecular property prediction. In: Proceedings of the 10th ACM international conference on bioinformatics, computational biology and health informatics. 429–436.

[54] Honda S, Shi S, Ueda HR (2019) Smiles transformer: Pre-trained molecular fingerprint for low data drug discovery. arXiv preprint arXiv*.*191104738.

[55] Irwin R, Dimitriadis S, He J, Bjerrum EJ (2022) Chemformer: a pre-trained transformer for computational chemistry. Mach Learn Sci Technol 3(1):015022

[56] Osipenko S, Botashev K, Nikolaev E, Kostyukevich Y (2021) Transfer learning for small molecule retention predictions. J Chromatogr A 1644:462119 33845426 10.1016/j.chroma.2021.462119

[57] Wigh DS, Goodman JM, Lapkin AA (2022) A review of molecular representation in the age of machine learning. Wiley Interdiscip Rev Comput Mol Sci 12(5):e1603

[58] Zhang X-M, Liang L, Liu L, Tang M-J (2021) Graph neural networks and their current applications in bioinformatics. Front Genet 12:690049 34394185 10.3389/fgene.2021.690049 PMC8360394

[59] Gori M, Monfardini G, Scarselli F (2005) A new model for learning in graph domains. In: Proceedings 2005 IEEE International Joint Conference on Neural Networks, 2005. IEEE: 729–734.

[60] Wieder O, Kohlbacher S, Kuenemann M, Garon A, Ducrot P, Seidel T, Langer T (2020) A compact review of molecular property prediction with graph neural networks. Drug Discov Today Technol 37:1–12 34895648 10.1016/j.ddtec.2020.11.009

[61] Kipf TN, Welling M (2016) Semi-supervised classification with graph convolutional networks. arXiv preprint arXiv:160902907.

[62] Fang X, Liu L, Lei J, He D, Zhang S, Zhou J, Wang F, Wu H, Wang H (2022) Geometry-enhanced molecular representation learning for property prediction. Nat Mach Intell 4(2):127–134

[63] Xu K, Hu W, Leskovec J, Jegelka S (2018) How powerful are graph neural networks? arXiv preprint arXiv:181000826.

[64] Sun R, Dai H, Yu AW (2022) Does GNN pretraining help molecular representation? Adv Neural Inf Process Syst 35:12096–12109

[65] Schlichtkrull M, Kipf TN, Bloem P, Van Den Berg R, Titov I, Welling M (2018) Modeling relational data with graph convolutional networks. In: The Semantic Web: 15th International Conference, ESWC 2018, Heraklion, Crete, Greece, June 3–7, 2018, Proceedings 15. Springer: 593–607.

[66] Gilmer J, Schoenholz SS, Riley PF, Vinyals O, Dahl GE (2017) Neural Message Passing for Quantum Chemistry. In: Proceedings of the 34th International Conference on Machine Learning; Proceedings of Machine Learning Research: Edited by Doina P, Yee Whye T. PMLR. 1263--1272.

[67] Vinyals O, Bengio S, Kudlur M (2015) Order matters: sequence to sequence for sets. arXiv preprint arXiv:151106391.

[68] Ramsundar B, Eastman P, Walters P, Pande V (2019) Deep learning for the life sciences: applying deep learning to genomics, microscopy, drug discovery, and more. O’Reilly Media Inc, Sebastopol

[69] Keras implementation of MPNN. https://keras.io/examples/graph/mpnn-molecular-graphs/#predicting.

[70] Osipenko S, Nikolaev E, Kostyukevich Y (2022) Retention time prediction with message-passing neural networks. Separations 9(10):291

[71] Pocha A, Danel T, Podlewska S, Tabor J, Maziarka Ł (2021) Comparison of atom representations in graph neural networks for molecular property prediction. In: 2021 International Joint Conference on Neural Networks (IJCNN). IEEE: 1–8.

[72] Wolfer AM, Lozano S, Umbdenstock T, Croixmarie V, Arrault A, Vayer P (2016) UPLC–MS retention time prediction: a machine learning approach to metabolite identification in untargeted profiling. Metabolomics 12(1):8

[73] Bonini P, Kind T, Tsugawa H, Barupal DK, Fiehn O (2020) Retip: retention time prediction for compound annotation in untargeted metabolomics. Anal Chem 92(11):7515–7522 32390414 10.1021/acs.analchem.9b05765 PMC8715951

[74] Kanehisa M, Furumichi M, Sato Y, Kawashima M, Ishiguro-Watanabe M (2023) KEGG for taxonomy-based analysis of pathways and genomes. Nucleic Acids Res 51(D1):D587–D592 36300620 10.1093/nar/gkac963 PMC9825424

[75] Tsugawa H, Cajka T, Kind T, Ma Y, Higgins B, Ikeda K, Kanazawa M, VanderGheynst J, Fiehn O, Arita M (2015) MS-DIAL: data-independent MS/MS deconvolution for comprehensive metabolome analysis. Nat Methods 12(6):523–526 25938372 10.1038/nmeth.3393 PMC4449330

[76] Tsugawa H, Kind T, Nakabayashi R, Yukihira D, Tanaka W, Cajka T, Saito K, Fiehn O, Arita M (2016) Hydrogen rearrangement rules: computational MS/MS fragmentation and structure elucidation using MS-FINDER software. Anal Chem 88(16):7946–7958 27419259 10.1021/acs.analchem.6b00770 PMC7063832

[77] Aicheler F, Li J, Hoene M, Lehmann R, Xu G, Kohlbacher O (2015) Retention time prediction improves identification in nontargeted lipidomics approaches. Anal Chem 87(15):7698–7704 26145158 10.1021/acs.analchem.5b01139

[78] Low DY, Micheau P, Koistinen VM, Hanhineva K, Abrankó L, Rodriguez-Mateos A, da Silva AB, van Poucke C, Almeida C, Andres-Lacueva C (2021) Data sharing in PredRet for accurate prediction of retention time: application to plant food bioactive compounds. Food Chem 357:129757 33872868 10.1016/j.foodchem.2021.129757

[79] Wu Z, Ramsundar B, Feinberg EN, Gomes J, Geniesse C, Pappu AS, Leswing K, Pande V (2018) MoleculeNet: a benchmark for molecular machine learning. Chem Sci 9(2):513–530 29629118 10.1039/c7sc02664a PMC5868307

[80] Wang B (2023) RT-Tranformer: retention time prediction for metabolite annotation to assist in metabolite identification. ChemRxiv. 10.26434/chemrxiv-2023-pf268-v2 10.1093/bioinformatics/btae084 PMC10914443 38402516

[81] Osipenko S, Bashkirova I, Sosnin S, Kovaleva O, Fedorov M, Nikolaev E, Kostyukevich Y (2020) Machine learning to predict retention time of small molecules in nano-HPLC. Anal Bioanal Chem 412:7767–7776 32860519 10.1007/s00216-020-02905-0

[82] Hall LM, Hill DW, Bugden K, Cawley S, Hall LH, Chen M-H, Grant DF (2018) Development of a reverse phase HPLC retention index model for nontargeted metabolomics using synthetic compounds. J Chem Inf Model 58(3):591–604 29489351 10.1021/acs.jcim.7b00496 PMC8404481

[83] Wen Y, Talebi M, Amos RI, Szucs R, Dolan JW, Pohl CA, Haddad PR (2018) Retention prediction in reversed phase high performance liquid chromatography using quantitative structure-retention relationships applied to the Hydrophobic Subtraction Model. J Chromatogr A 1541:1–11 29454529 10.1016/j.chroma.2018.01.053

[84] Wen Y, Amos RI, Talebi M, Szucs R, Dolan JW, Pohl CA, Haddad PR (2018) Retention index prediction using quantitative structure–retention relationships for improving structure identification in nontargeted metabolomics. Anal Chem 90(15):9434–9440 29952550 10.1021/acs.analchem.8b02084

[85] McEachran AD, Mansouri K, Newton SR, Beverly BE, Sobus JR, Williams AJ (2018) A comparison of three liquid chromatography (LC) retention time prediction models. Talanta 182:371–379 29501166 10.1016/j.talanta.2018.01.022 PMC6066181

[86] Falchi F, Bertozzi SM, Ottonello G, Ruda GF, Colombano G, Fiorelli C, Martucci C, Bertorelli R, Scarpelli R, Cavalli A (2016) Kernel-based, partial least squares quantitative structure-retention relationship model for UPLC retention time prediction: a useful tool for metabolite identification. Anal Chem 88(19):9510–9517 27583774 10.1021/acs.analchem.6b02075

[87] Kumari P, Van Laethem T, Duroux D, Fillet M, Hubert P, Sacré P-Y, Hubert C (2023) A multi-target QSRR approach to model retention times of small molecules in RPLC. J Pharm Biomed Anal 236:115690 37688907 10.1016/j.jpba.2023.115690

[88] Liapikos T, Zisi C, Kodra D, Kademoglou K, Diamantidou D, Begou O, Pappa-Louisi A, Theodoridis G (2022) Quantitative structure retention relationship (QSRR) modelling for Analytes’ retention prediction in LC-HRMS by applying different Machine Learning algorithms and evaluating their performance. J Chromatogr B 1191:123132 10.1016/j.jchromb.2022.123132 35093854

[89] Souihi A, Mohai MP, Palm E, Malm L, Kruve A (2022) MultiConditionRT: predicting liquid chromatography retention time for emerging contaminants for a wide range of eluent compositions and stationary phases. J Chromatogr A 1666:462867 35139450 10.1016/j.chroma.2022.462867

[90] Pasin D, Mollerup CB, Rasmussen BS, Linnet K, Dalsgaard PW (2021) Development of a single retention time prediction model integrating multiple liquid chromatography systems: application to new psychoactive substances. Anal Chim Acta 1184:339035 34625246 10.1016/j.aca.2021.339035

[91] Kruve A, Kiefer K, Hollender J (2021) Benchmarking of the quantification approaches for the non-targeted screening of micropollutants and their transformation products in groundwater. Anal Bioanal Chem 413:1549–1559 33506334 10.1007/s00216-020-03109-2 PMC7921029

