# Supplementary material for: Insights into predicting small molecule retention times in liquid chromatography using deep learning
Source: J Cheminform. 2024 Oct 7;16:113. doi: 10.1186/s13321-024-00905-1 (PMC11460055; doi:10.1186/s13321-024-00905-1)
Supplement: Supplementary file 1 — Supplementary material 1. [file 13321_2024_905_MOESM1_ESM.docx]

Supplementary Table 1. The list of 32 small datasets assessed in this review from PredRet.

| FEM_long |
| --- |
| FEM_short |
| LIFE_new |
| LIFE_old |
| IPB_Halle |
| MTBLS20 |
| RIKEN |
| MTBLS4 |
| Eawag_XBridgeC18 |
| UniAthens_Acclaim |
| Cao_HILIC |
| FEM_long2 |
| Fang_HILIC1 |
| KI-GIAR zic-HILIC pH2.7 |
| KI_GIAR_zic_HILIC_pH2_7 |
| MTBLS20-LIUMIN |
| ZIC-pHILIC pH 9.9 |
| Bade_Publi |
| KWR_Publi |
| METLIN RP |
| Waters STA Forensic |
| CBM_TEST_F |
| CBM_Test_G |
| WORKPJ |
| MPE_IPK_Gatersleben |
| BfG_NTS_RP1 |
| UNNE-C18 shimadzu LC10/20 |
| semitargetedHSST3 |
| ACQUITY_UPLC_BEH_C18_150 mm_POS |
| FEM_long_JMM |
| Chen_Waters_SERI2019 |
| Chen_Waters_SERI2019_58PFAS |
